# (*S*)-*N*-[(1*S*,2*S*)-2-Benzyl­amino-1-(4-hy­droxy­phen­yl)-3-methyl­butyl]-1,1-di­methyl­ethane-2-sulfinamide

**DOI:** 10.1107/S1600536808031073

**Published:** 2008-10-09

**Authors:** Chun Shen

**Affiliations:** a Department of Chemistry, East China Normal University, 3663 Zhongshan Road, Shanghai 200062, People’s Republic of China

## Abstract

The title compound, C_22_H_32_N_2_O_2_S, was obtained by dehydroxy­lation and deacetyl­ation of 4-{(1*S*,2*S*)-2-(benzylhydroxy­amino)-3-methyl-1-[(*S*)-2-methyl­propane-2-sulfinylamino]but­yl}phenyl acetate, which was derived from reductive crosslinking of nitrone with *N*-*tert*-butane­sulfinyl­imine. The crystal structure shows that the mol­ecules are linked by O—H⋯O hydrogen bonds.

## Related literature

For general background on optically pure vicinal diamines, see: Bennai & Hanessian (1997 ▶); Kizirian (2008 ▶). For the synthesis of the starting material, see: Zhong *et al.* (2004 ▶).
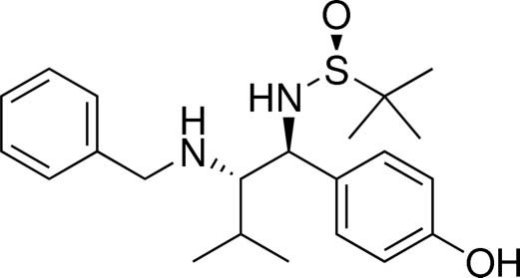


## Experimental

### 

#### Crystal data

C_22_H_32_N_2_O_2_S
*M* *_r_* = 388.56
Orthorhombic, 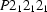

*a* = 9.7503 (9) Å
*b* = 12.1068 (11) Å
*c* = 19.6292 (18) Å
*V* = 2317.1 (4) Å^3^
*Z* = 4
Mo *K*α radiation
μ = 0.16 mm^−1^
*T* = 293 (2) K
0.45 × 0.40 × 0.29 mm

#### Data collection

Bruker SMART APEX CCD area-detector diffractometer
Absorption correction: multi-scan (*SADABS*; Bruker, 2001 ▶) *T*_min_ = 0.783, *T*_max_ = 1.000 (expected range = 0.749–0.956)
13756 measured reflections
5016 independent reflections
2765 reflections with *I* > 2σ(*I*)
*R*_int_ = 0.038

#### Refinement

*R*[*F* ^2^ > 2σ(*F* ^2^)] = 0.052
*wR*(*F* ^2^) = 0.130
*S* = 0.89
5016 reflections
261 parameters
3 restraints
H atoms treated by a mixture of independent and constrained refinement
Δρ_max_ = 0.17 e Å^−3^
Δρ_min_ = −0.16 e Å^−3^
Absolute structure: Flack (1983 ▶), with 2249 Friedel pairs
Flack parameter: 0.01 (10)

### 

Data collection: *SMART* (Bruker, 2001 ▶); cell refinement: *SAINT* (Bruker, 2001 ▶); data reduction: *SAINT*; program(s) used to solve structure: *SHELXS97* (Sheldrick, 2008 ▶); program(s) used to refine structure: *SHELXL97* (Sheldrick, 2008 ▶); molecular graphics: *SHELXTL* (Sheldrick, 2008 ▶); software used to prepare material for publication: *SHELXTL*.

## Supplementary Material

Crystal structure: contains datablocks I, global. DOI: 10.1107/S1600536808031073/bv2109sup1.cif

Structure factors: contains datablocks I. DOI: 10.1107/S1600536808031073/bv2109Isup2.hkl

Additional supplementary materials:  crystallographic information; 3D view; checkCIF report

## Figures and Tables

**Table 1:** Hydrogen-bond geometry (Å, °)

*D*—H⋯*A*	*D*—H	H⋯*A*	*D*⋯*A*	*D*—H⋯*A*
O1—H1*B*⋯O2^i^	0.828 (19)	1.82 (2)	2.647 (4)	173 (5)

Symmetry code: (i) 
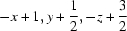
.

**Fig. 1.**
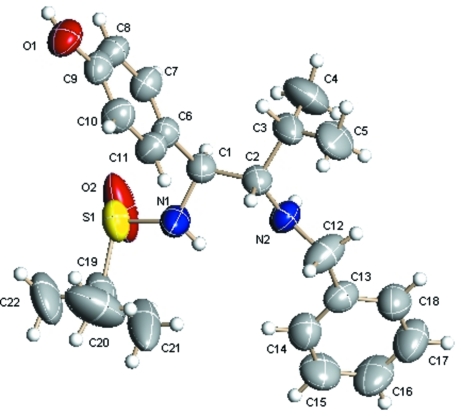
Plot of C22H32N2O2S at the 50% probability level. H atoms are drawn as spheres of arbitrary radius.

**Fig. 2.**
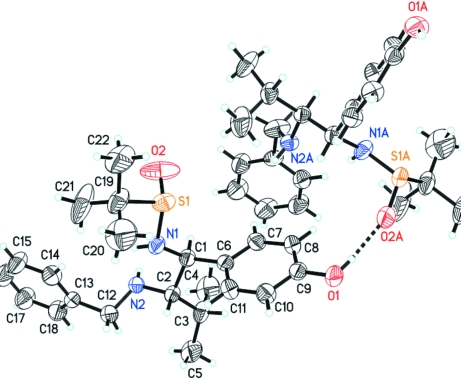
Hydrogen bonding in the title compound. Symmetry codes: (i) -x + 1, y + 1/2, -z + 3/2.

**Fig. 3.**
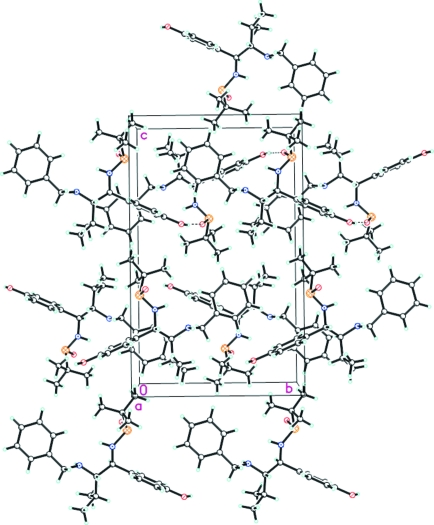
Molecular packing plot, viewed along a axis. Hydrogen bond interactions are shown as dashed lines.

## supplementary crystallographic information

### Comment

Optically pure vicinal diamines are important molecules due to their special structures. Among them, a lot of compounds have been used as catalysts in asymmetric reactions (Bennai & Hanessian, 1997; Kizirian, 2008). In our study of vicinal diamines, we prepared (*S*)-*N*-((1*S*,2*S*)-2-(benzylamino)-1-(4-hydroxyphenyl) -3-methylbutyl)-2-methylpropane-2-sulfinamide through dehydroxylation and deacetylation of acetic acid 4-[(1*S*,2*S*)-2(benzyl-hydroxy-amino)-3-methyl-1- ((*S*)-2-methyl-propane-2-sulfinylamino)-butyl]-phenyl ester which was prepared according to the reported procedure (Zhong *et al.*, 2004). Here, we report its crystal structure. The molecules are linked by a strong intermolecular O1—H1B···O2^i^ hydrogen interaction. The molecular packing for the compound is shown in Fig. 3, where hydrogen bond interactions are shown as dashed lines. The two benzene rings are almost perpendicular to each other, making a diheral angle of 84.12(0.12)°. The molecule exists in a *trans* configuration with an N1—C1—C2—N2 torsion angle of 55.6 (3)°. The absolute configuration was known from the starting chiral material and is confirmed by this X-ray analysis.

### Experimental

A mixture of Cu(II) acetate (18 mg, 0.1 mmol), zinc powder (324 mg, 5.0 mmol) and acetic acid (2 ml) was stirred for 15 minutes under a nitrogen atmosphere at room temperature. Then a mixture of acetic acid (2 ml), distilled water (0.7 ml) and acetic acid 4-[(1*S*,2*S*)-2(benzyl-hydroxy-amino)-3-methyl -1-((*S*)-2-methyl-propane-2-sulfinylamino)-butyl]-phenyl ester (Zhong *et al.*, 2004) (446 mg, 1.0 mmol) was added. The resulting mixture was heated to 343 K and stirred for an hour. After cooling to room temperature, ethylene diamine tetraacetic acid disodium salt (0.5 g) was added and stirred for 10 minutes. Aqueous KOH (3*N*) solution was then added to adjust the mixture to a pH value of 10. The resulting solution was extracted with ethyl acetate, and the combined organic layers were washed with saturated aqueous ethylene diamine tetraacetic acid disodium salt and brine successively. Concentrated under reduced pressure, the crude product was dissolved in MeOH (10 ml) and saturated aqueous NaHCO_3_ (10 ml) was added. The mixture was stirred for 12 h at room temperature and then MeOH was removed under reduced pressure. The crude solid was redissolved in CH_2_Cl_2_, washed with brine and dried with anhydrous Na_2_SO_4_. After silica gel chromatography, the pure product was obtained as a white solid (yield 77% over two steps). Suitable crystals for the X-ray diffraction experiment were obtained by recrystalization from hexane/CH_2_Cl_2_ (3:1). Spectroscopic data: ^1^H NMR (300 MHz, CDCl_3_) δ 7.36–7.24 (m, 5H), 7.11 (d, *J* = 6.0 Hz, 2H), 6.80 (d, *J *= 5.7 Hz, 2H), 5.49 (s, 1H), 4.02 (m, 2H), 3.77 (d, *J* = 13.2 Hz, 1H), 2.63(d, *J *= 9.3 Hz, 1H), 1.72 (m, 2H), 1.14 (s, 9H), 0.95 (d, *J* = 4.5 Hz, 3H), 0.77 (d, *J* = 4.5 Hz, 3H). ESI-MS (m/*z*): 389(*M*+H^+^).

### Refinement

The hydrogen atoms were generated geometrically (C—H = 0.93, 0.98, 0.97 or 0.96 Å for phenyl, tertiary, methylene or methyl H atoms respectively. The H atoms attached to O and N were refined isotropically. The displacement parameters of methyl H atoms were set to 1.5 times *U*_eq_ of the equivalent isotropic displacement parameters of their parent atoms (1.2 times for H atoms attached to phenyl, tertiary, or methylene C atoms).

### Crystal data

**Table:**

C_22_H_32_N_2_O_2_S	*F*(000) = 840
*M**_r_* = 388.56	*D*_x_ = 1.114 Mg m^−^^3^
Orthorhombic, *P*2_1_2_1_2_1_	Mo *K*α radiation, λ = 0.71073 Å
Hall symbol: P 2ac 2ab	Cell parameters from 2613 reflections
*a* = 9.7503 (9) Å	θ = 4.7–40.0°
*b* = 12.1068 (11) Å	µ = 0.16 mm^−^^1^
*c* = 19.6292 (18) Å	*T* = 293 K
*V* = 2317.1 (4) Å^3^	Prismatic, colourless
*Z* = 4	0.45 × 0.40 × 0.29 mm

### Data collection

**Table:**

Bruker SMART APEX CCD area-detector diffractometer	5016 independent reflections
Radiation source: fine-focus sealed tube	2765 reflections with *I* > 2σ(*I*)
graphite	*R*_int_ = 0.038
φ and ω scans	θ_max_ = 27.0°, θ_min_ = 2.0°
Absorption correction: multi-scan (SADABS; Bruker, 2001)	*h* = −11→12
*T*_min_ = 0.784, *T*_max_ = 1.000	*k* = −14→15
13756 measured reflections	*l* = −23→25

### Refinement

**Table:**

Refinement on *F*^2^	Secondary atom site location: difference Fourier map
Least-squares matrix: full	Hydrogen site location: inferred from neighbouring sites
*R*[*F*^2^ > 2σ(*F*^2^)] = 0.052	H atoms treated by a mixture of independent and constrained refinement
*wR*(*F*^2^) = 0.130	*w* = 1/[σ^2^(*F*_o_^2^) + (0.0628*P*)^2^] where *P* = (*F*_o_^2^ + 2*F*_c_^2^)/3
*S* = 0.89	(Δ/σ)_max_ = 0.001
5016 reflections	Δρ_max_ = 0.17 e Å^−^^3^
261 parameters	Δρ_min_ = −0.16 e Å^−^^3^
3 restraints	Absolute structure: Flack (1983), with 2249 Friedel pairs
Primary atom site location: structure-invariant direct methods	Flack parameter: 0.01 (10)

### Special details

**Table:**

Geometry. All e.s.d.'s (except the e.s.d. in the dihedral angle between two l.s. planes) are estimated using the full covariance matrix. The cell e.s.d.'s are taken into account individually in the estimation of e.s.d.'s in distances, angles and torsion angles; correlations between e.s.d.'s in cell parameters are only used when they are defined by crystal symmetry. An approximate (isotropic) treatment of cell e.s.d.'s is used for estimating e.s.d.'s involving l.s. planes.
Refinement. Refinement of *F*^2^ against ALL reflections. The weighted *R*-factor *wR* and goodness of fit *S* are based on *F*^2^, conventional *R*-factors *R* are based on *F*, with *F* set to zero for negative *F*^2^. The threshold expression of *F*^2^ > σ(*F*^2^) is used only for calculating *R*-factors(gt) *etc*. and is not relevant to the choice of reflections for refinement. *R*-factors based on *F*^2^ are statistically about twice as large as those based on *F*, and *R*- factors based on ALL data will be even larger.

### Fractional atomic coordinates and isotropic or equivalent isotropic displacement parameters (Å^2^)

**Table:**

	*x*	*y*	*z*	*U*_iso_*/*U*_eq_	
S1	0.65403 (9)	0.95415 (7)	0.85995 (4)	0.0833 (3)	
O1	0.7047 (3)	1.29113 (19)	0.61688 (11)	0.0852 (7)	
O2	0.5196 (3)	0.9143 (2)	0.88218 (16)	0.1612 (15)	
N1	0.7265 (3)	0.8691 (2)	0.80538 (11)	0.0728 (7)	
N2	0.7310 (3)	0.6729 (2)	0.73797 (12)	0.0631 (6)	
C1	0.6665 (3)	0.8654 (2)	0.73606 (12)	0.0632 (7)	
H1	0.5697	0.8445	0.7396	0.076*	
C2	0.7439 (3)	0.7739 (2)	0.69788 (12)	0.0605 (7)	
H2	0.8412	0.7943	0.6988	0.073*	
C3	0.7030 (4)	0.7655 (3)	0.62223 (14)	0.0791 (9)	
H3	0.7041	0.8411	0.6044	0.095*	
C4	0.5602 (5)	0.7234 (4)	0.61216 (19)	0.1376 (17)	
H4A	0.5316	0.7378	0.5662	0.206*	
H4B	0.4993	0.7602	0.6432	0.206*	
H4C	0.5580	0.6454	0.6206	0.206*	
C5	0.8053 (5)	0.7010 (3)	0.58036 (15)	0.1184 (14)	
H5A	0.8967	0.7229	0.5926	0.178*	
H5B	0.7904	0.7156	0.5329	0.178*	
H5C	0.7939	0.6235	0.5890	0.178*	
C6	0.6761 (3)	0.9788 (2)	0.70327 (12)	0.0590 (7)	
C7	0.5600 (3)	1.0341 (3)	0.68355 (14)	0.0728 (8)	
H7	0.4750	1.0008	0.6898	0.087*	
C8	0.5659 (3)	1.1384 (3)	0.65456 (15)	0.0750 (8)	
H8	0.4855	1.1745	0.6420	0.090*	
C9	0.6911 (3)	1.1884 (2)	0.64437 (14)	0.0677 (7)	
C10	0.8081 (3)	1.1331 (3)	0.66348 (15)	0.0777 (8)	
H10	0.8933	1.1658	0.6568	0.093*	
C11	0.7999 (3)	1.0292 (3)	0.69266 (14)	0.0736 (8)	
H11	0.8801	0.9929	0.7053	0.088*	
C12	0.8389 (4)	0.5915 (3)	0.73358 (17)	0.1013 (12)	
H12A	0.9268	0.6284	0.7375	0.122*	
H12B	0.8351	0.5565	0.6892	0.122*	
C13	0.8291 (3)	0.5047 (3)	0.78733 (14)	0.0700 (8)	
C14	0.8323 (3)	0.5296 (3)	0.85489 (16)	0.0841 (8)	
H14	0.8391	0.6032	0.8680	0.101*	
C15	0.8259 (4)	0.4497 (4)	0.90415 (16)	0.1048 (11)	
H15	0.8300	0.4686	0.9500	0.126*	
C16	0.8133 (5)	0.3416 (4)	0.8847 (2)	0.1146 (14)	
H16	0.8085	0.2867	0.9177	0.138*	
C17	0.8076 (5)	0.3137 (3)	0.8174 (2)	0.1120 (13)	
H17	0.7971	0.2404	0.8043	0.134*	
C18	0.8176 (4)	0.3956 (3)	0.76973 (17)	0.0913 (10)	
H18	0.8166	0.3766	0.7238	0.110*	
C19	0.7700 (5)	0.9328 (3)	0.93164 (15)	0.1022 (13)	
C20	0.9065 (5)	0.9748 (6)	0.9100 (3)	0.184 (2)	
H20A	0.9624	0.9878	0.9495	0.276*	
H20B	0.8952	1.0427	0.8853	0.276*	
H20C	0.9502	0.9212	0.8813	0.276*	
C21	0.7701 (9)	0.8136 (3)	0.95298 (19)	0.187 (4)	
H21A	0.8172	0.7703	0.9194	0.325*	
H21B	0.6773	0.7880	0.9571	0.325*	
H21C	0.8158	0.8064	0.9961	0.325*	
C22	0.7108 (6)	1.0067 (3)	0.98828 (17)	0.1491 (19)	
H22A	0.7718	1.0066	1.0267	0.224*	
H22B	0.6228	0.9788	1.0020	0.224*	
H22C	0.7007	1.0807	0.9715	0.224*	
H1A	0.738 (3)	0.8013 (15)	0.8199 (13)	0.065 (9)*	
H2A	0.651 (2)	0.645 (3)	0.7369 (16)	0.094 (12)*	
H1B	0.634 (3)	1.329 (3)	0.614 (2)	0.15 (2)*	

### Atomic displacement parameters (Å^2^)

**Table:**

	*U*^11^	*U*^22^	*U*^33^	*U*^12^	*U*^13^	*U*^23^
S1	0.1059 (7)	0.0689 (5)	0.0750 (5)	−0.0119 (5)	0.0166 (4)	−0.0222 (4)
O1	0.1074 (19)	0.0627 (15)	0.0853 (15)	0.0058 (14)	0.0131 (13)	0.0169 (12)
O2	0.135 (2)	0.174 (3)	0.175 (3)	−0.068 (2)	0.076 (2)	−0.113 (2)
N1	0.1051 (19)	0.0633 (17)	0.0501 (13)	0.0144 (15)	0.0058 (12)	0.0019 (13)
N2	0.0687 (18)	0.0526 (15)	0.0680 (15)	0.0056 (13)	0.0095 (12)	0.0088 (12)
C1	0.0726 (18)	0.0592 (17)	0.0579 (15)	0.0020 (15)	−0.0025 (15)	0.0007 (14)
C2	0.0737 (18)	0.0556 (17)	0.0524 (14)	−0.0021 (14)	0.0000 (14)	0.0042 (13)
C3	0.119 (3)	0.0614 (19)	0.0570 (17)	−0.0054 (19)	−0.0165 (17)	0.0032 (15)
C4	0.151 (4)	0.160 (4)	0.101 (3)	−0.011 (3)	−0.056 (3)	−0.019 (3)
C5	0.173 (4)	0.130 (3)	0.0521 (17)	0.028 (3)	−0.003 (2)	−0.018 (2)
C6	0.0635 (17)	0.0550 (17)	0.0584 (14)	0.0033 (15)	0.0039 (13)	−0.0034 (13)
C7	0.072 (2)	0.065 (2)	0.0817 (19)	0.0047 (16)	0.0077 (15)	0.0019 (18)
C8	0.082 (2)	0.065 (2)	0.077 (2)	0.0141 (17)	0.0021 (17)	0.0095 (17)
C9	0.088 (2)	0.0566 (18)	0.0590 (15)	0.0067 (17)	0.0086 (16)	−0.0019 (15)
C10	0.074 (2)	0.070 (2)	0.090 (2)	−0.0025 (17)	−0.0012 (17)	0.0027 (18)
C11	0.073 (2)	0.063 (2)	0.0848 (19)	0.0046 (16)	−0.0082 (15)	0.0026 (17)
C12	0.113 (3)	0.090 (2)	0.101 (2)	0.036 (2)	0.036 (2)	0.032 (2)
C13	0.0714 (19)	0.074 (2)	0.0646 (17)	0.0123 (17)	0.0114 (15)	0.0132 (16)
C14	0.094 (2)	0.072 (2)	0.086 (2)	−0.0030 (19)	0.0030 (19)	−0.003 (2)
C15	0.142 (3)	0.112 (3)	0.0610 (18)	−0.002 (3)	0.002 (2)	0.011 (2)
C16	0.164 (4)	0.085 (3)	0.094 (3)	0.016 (3)	0.016 (3)	0.030 (2)
C17	0.164 (4)	0.071 (2)	0.101 (3)	0.012 (3)	0.019 (3)	0.009 (2)
C18	0.116 (3)	0.079 (3)	0.079 (2)	0.009 (2)	0.000 (2)	−0.0023 (19)
C19	0.181 (4)	0.070 (2)	0.0555 (17)	−0.014 (2)	−0.002 (2)	−0.0059 (17)
C20	0.140 (4)	0.283 (8)	0.130 (4)	−0.007 (5)	−0.052 (3)	−0.018 (5)
C21	0.309 (12)	0.076 (3)	0.065 (2)	0.030 (4)	−0.043 (4)	−0.003 (2)
C22	0.249 (6)	0.111 (3)	0.087 (2)	−0.012 (3)	0.005 (3)	−0.047 (2)

### Geometric parameters (Å, °)

**Table:**

S1—O2	1.463 (3)	C9—C10	1.374 (4)
S1—N1	1.645 (2)	C10—C11	1.385 (4)
S1—C19	1.824 (4)	C10—H10	0.9300
O1—C9	1.363 (3)	C11—H11	0.9300
O1—H1B	0.828 (19)	C12—C13	1.492 (4)
N1—C1	1.481 (3)	C12—H12A	0.9700
N1—H1A	0.875 (17)	C12—H12B	0.9700
N2—C12	1.443 (4)	C13—C14	1.360 (4)
N2—C2	1.460 (3)	C13—C18	1.370 (4)
N2—H2A	0.847 (18)	C14—C15	1.369 (4)
C1—C6	1.520 (4)	C14—H14	0.9300
C1—C2	1.536 (4)	C15—C16	1.368 (5)
C1—H1	0.9800	C15—H15	0.9300
C2—C3	1.541 (4)	C16—C17	1.364 (5)
C2—H2	0.9800	C16—H16	0.9300
C3—C4	1.496 (5)	C17—C18	1.368 (5)
C3—C5	1.510 (5)	C17—H17	0.9300
C3—H3	0.9800	C18—H18	0.9300
C4—H4A	0.9600	C19—C20	1.487 (6)
C4—H4B	0.9600	C19—C21	1.502 (5)
C4—H4C	0.9600	C19—C22	1.539 (5)
C5—H5A	0.9600	C20—H20A	0.9600
C5—H5B	0.9600	C20—H20B	0.9600
C5—H5C	0.9600	C20—H20C	0.9600
C6—C11	1.368 (4)	C21—H21A	0.9600
C6—C7	1.371 (4)	C21—H21B	0.9600
C7—C8	1.386 (4)	C21—H21C	0.9600
C7—H7	0.9300	C22—H22A	0.9600
C8—C9	1.378 (4)	C22—H22B	0.9600
C8—H8	0.9300	C22—H22C	0.9600
			
O2—S1—N1	111.86 (14)	C9—C10—H10	119.8
O2—S1—C19	106.2 (2)	C11—C10—H10	119.8
N1—S1—C19	98.49 (15)	C6—C11—C10	121.2 (3)
C9—O1—H1B	117 (3)	C6—C11—H11	119.4
C1—N1—S1	116.59 (19)	C10—C11—H11	119.4
C1—N1—H1A	108.7 (18)	N2—C12—C13	113.1 (2)
S1—N1—H1A	115.4 (18)	N2—C12—H12A	109.0
C12—N2—C2	118.5 (2)	C13—C12—H12A	109.0
C12—N2—H2A	114 (2)	N2—C12—H12B	109.0
C2—N2—H2A	113 (2)	C13—C12—H12B	109.0
N1—C1—C6	109.7 (2)	H12A—C12—H12B	107.8
N1—C1—C2	106.0 (2)	C14—C13—C18	117.5 (3)
C6—C1—C2	114.5 (2)	C14—C13—C12	122.1 (3)
N1—C1—H1	108.8	C18—C13—C12	120.4 (3)
C6—C1—H1	108.8	C13—C14—C15	122.1 (3)
C2—C1—H1	108.8	C13—C14—H14	119.0
N2—C2—C1	107.4 (2)	C15—C14—H14	119.0
N2—C2—C3	116.2 (2)	C16—C15—C14	118.8 (3)
C1—C2—C3	113.0 (2)	C16—C15—H15	120.6
N2—C2—H2	106.5	C14—C15—H15	120.6
C1—C2—H2	106.5	C17—C16—C15	120.7 (3)
C3—C2—H2	106.5	C17—C16—H16	119.6
C4—C3—C5	111.5 (3)	C15—C16—H16	119.6
C4—C3—C2	113.0 (3)	C16—C17—C18	118.7 (4)
C5—C3—C2	112.8 (3)	C16—C17—H17	120.7
C4—C3—H3	106.3	C18—C17—H17	120.7
C5—C3—H3	106.3	C17—C18—C13	122.2 (3)
C2—C3—H3	106.3	C17—C18—H18	118.9
C3—C4—H4A	109.5	C13—C18—H18	118.9
C3—C4—H4B	109.5	C20—C19—C21	114.1 (5)
H4A—C4—H4B	109.5	C20—C19—C22	110.1 (4)
C3—C4—H4C	109.5	C21—C19—C22	110.9 (3)
H4A—C4—H4C	109.5	C20—C19—S1	106.6 (3)
H4B—C4—H4C	109.5	C21—C19—S1	110.6 (3)
C3—C5—H5A	109.5	C22—C19—S1	104.0 (3)
C3—C5—H5B	109.5	C19—C20—H20A	109.5
H5A—C5—H5B	109.5	C19—C20—H20B	109.5
C3—C5—H5C	109.5	H20A—C20—H20B	109.5
H5A—C5—H5C	109.5	C19—C20—H20C	109.5
H5B—C5—H5C	109.5	H20A—C20—H20C	109.5
C11—C6—C7	117.9 (3)	H20B—C20—H20C	109.5
C11—C6—C1	121.4 (3)	C19—C21—H21A	109.5
C7—C6—C1	120.7 (3)	C19—C21—H21B	109.5
C6—C7—C8	121.8 (3)	H21A—C21—H21B	109.5
C6—C7—H7	119.1	C19—C21—H21C	109.5
C8—C7—H7	119.1	H21A—C21—H21C	109.5
C9—C8—C7	119.8 (3)	H21B—C21—H21C	109.5
C9—C8—H8	120.1	C19—C22—H22A	109.5
C7—C8—H8	120.1	C19—C22—H22B	109.5
O1—C9—C10	118.2 (3)	H22A—C22—H22B	109.5
O1—C9—C8	123.0 (3)	C19—C22—H22C	109.5
C10—C9—C8	118.8 (3)	H22A—C22—H22C	109.5
C9—C10—C11	120.5 (3)	H22B—C22—H22C	109.5
			
O2—S1—N1—C1	72.0 (3)	O1—C9—C10—C11	179.0 (2)
C19—S1—N1—C1	−176.7 (2)	C8—C9—C10—C11	−0.2 (4)
S1—N1—C1—C6	60.5 (3)	C7—C6—C11—C10	0.6 (4)
S1—N1—C1—C2	−175.33 (19)	C1—C6—C11—C10	−179.2 (2)
C12—N2—C2—C1	−151.5 (3)	C9—C10—C11—C6	−0.1 (5)
C12—N2—C2—C3	80.9 (4)	C2—N2—C12—C13	167.6 (3)
N1—C1—C2—N2	55.6 (3)	N2—C12—C13—C14	−59.0 (5)
C6—C1—C2—N2	176.7 (2)	N2—C12—C13—C18	121.6 (4)
N1—C1—C2—C3	−174.9 (2)	C18—C13—C14—C15	0.7 (5)
C6—C1—C2—C3	−53.8 (3)	C12—C13—C14—C15	−178.8 (3)
N2—C2—C3—C4	55.6 (4)	C13—C14—C15—C16	−1.2 (6)
C1—C2—C3—C4	−69.3 (4)	C14—C15—C16—C17	0.2 (7)
N2—C2—C3—C5	−72.1 (4)	C15—C16—C17—C18	1.3 (7)
C1—C2—C3—C5	163.1 (3)	C16—C17—C18—C13	−1.9 (6)
N1—C1—C6—C11	60.3 (3)	C14—C13—C18—C17	0.9 (5)
C2—C1—C6—C11	−58.7 (3)	C12—C13—C18—C17	−179.6 (3)
N1—C1—C6—C7	−119.5 (3)	O2—S1—C19—C20	−179.0 (3)
C2—C1—C6—C7	121.5 (3)	N1—S1—C19—C20	65.2 (3)
C11—C6—C7—C8	−0.9 (4)	O2—S1—C19—C21	56.4 (4)
C1—C6—C7—C8	178.9 (2)	N1—S1—C19—C21	−59.4 (4)
C6—C7—C8—C9	0.6 (4)	O2—S1—C19—C22	−62.7 (3)
C7—C8—C9—O1	−179.2 (3)	N1—S1—C19—C22	−178.5 (3)
C7—C8—C9—C10	−0.1 (4)		

### Hydrogen-bond geometry (Å, °)

**Table:**

*D*—H···*A*	*D*—H	H···*A*	*D*···*A*	*D*—H···*A*
O1—H1B···O2^i^	0.83 (2)	1.82 (2)	2.647 (4)	173 (5)

Symmetry codes: (i) −*x*+1, *y*+1/2, −*z*+3/2.
